# Cost-effectiveness analysis of CDK4/6 inhibitors in the second-line treatment for HR+/HER2− advanced or metastatic breast cancer

**DOI:** 10.3389/fphar.2025.1540088

**Published:** 2025-09-08

**Authors:** Kaixuan Wang, Shixian Liu, Shunping Li, Jie Gao

**Affiliations:** ^1^ Shandong Provincial Maternal and Child Health Care Hospital Affiliated to Qingdao University, Jinan, China; ^2^ Centre for Health Management and Policy Research, School of Public Health, Cheeloo College of Medicine, Shandong University, Jinan, China

**Keywords:** cost-effectiveness, HR+/HER2− advanced or metastatic breast cancer, CDK4/6 inhibitors, abemaciclib, palbociclib, ribociclib, fulvestrant

## Abstract

**Objective:**

The aim of this study was to compare the cost-effectiveness of various CDK4/6 inhibitors plus fulvestrant with fulvestrant monotherapy in the second-line treatment for patients with HR+/HER2− advanced or metastatic breast cancer from the Chinese healthcare system perspective.

**Methods:**

A partitioned survival model was established to investigate the total costs, quality-adjusted life-years (QALYs), and incremental cost-effectiveness ratio (ICER) over a 10-year lifetime horizon. Clinical data was derived from the MONARCH 2 trial, PALOMA 3 trial and MONALEESA 3 trial; direct medical costs and utilities were acquired from local charges and published literature. Scenario, one-way and probabilistic sensitivity analyses were performed to test the robustness of the model.

**Results:**

In the base-case analysis, abemaciclib plus fulvestrant, palbociclib plus fulvestrant, ribociclib plus fulvestrant resulted in ICERs of $3,636.51/QALY, $1,256.32/QALY, and $39,654.78/QALY, respectively, compared with fulvestrant monotherapy. In the pairwise comparison between three CDK4/6 inhibitors, abemaciclib plus fulvestrant was the most cost-effectiveness treatment option. One-way sensitivity analysis showed that the proportion of subsequent treatment, utility values of progression-free survival (PFS), cost of best supportive care had a significant impact on ICER. Probabilistic sensitivity analysis demonstrated that abemaciclib plus fulvestrant achieved an overwhelming superiority with a 99.82% probability to be the most cost-effective strategy in China at the current price and willingness-to-pay threshold.

**Conclusion:**

From the perspective of Chinese healthcare system, abemaciclib plus fulvestrant represented the optimal regimen as the second-line treatment for HR+/HER2− advanced or metastatic breast cancer.

## Introduction

Breast cancer is the most commonly diagnosed cancer and the fifth leading cause of cancer death worldwide in 2020, with an estimated 2,261,419 new cases and 684,996 deaths (Sung et al., 2021). In China, there were around 420,000 new cases of breast cancer, resulting in approximately 120,000 deaths in the same year (Sung et al., 2021). Nearly, 6% of patients have metastatic disease at the time of diagnosis and approximately half of the patients with primary breast cancer will progress to the metastatic stage (Lu et al., 2009). Hormone receptor-positive (HR+)/human epidermal growth factor receptor 2-negative (HER2−), the most common molecular subtypes of breast cancer, accounts for 70% of all primary breast cancers (Howlader et al., 2014).

Although endocrine therapy is the main treatment for HR+/HER2− advanced or metastatic breast cancer, resistance to endocrine therapy and subsequent disease progression remains a major challenge (Milani et al., 2014). The use of cyclin-dependent kinases 4 and 6 (CDK4/6) inhibitors in combination with further endocrine therapy have emerged as a standard-of-care treatment for patients with HR+/HER2− advanced or metastatic breast cancer to overcome endocrine resistance and improve their clinical benefits. Inhibition of CDK4/6 leads to cell cycle arrest, thus inhibiting the DNA synthesis and proliferation of tumor cells (Xu et al., 2017). Based on MONARCH 2, PALOMA 3 and MONALEESA 3 trials, abemaciclib, palbociclib and ribociclib added to fulvestrant have shown clinically meaningful efficacy and manageable safety profiles in patients with endocrine-sensitive and endocrine-resistant HR+/HER2− advanced or metastatic breast cancer (Cristofanilli et al., 2022; Slamon et al., 2021; Sledge et al., 2020). As a result, these three CDK4/6 inhibitors mentioned above have been in succession approved by the National Medical Products Administration and recommended by the Guidelines of Chinese Society of Clinical Oncology (CSCO, 2024), and officially entered the National Reimbursement Drug List (NRDL) in 2024 for patients with HR+/HER2− advanced or metastatic breast cancer.

As multiple treatment options were available for HR+/HER2− advanced or metastatic breast cancer, there was a need to determine the optimal regimen, which was crucial for the clinical oncologists and healthcare policymakers. China faces significant healthcare resource constraints, and cost-effectiveness analysis plays a pivotal role in price negotiations and policy decision-making in drug reimbursement (Liu et al., 2025). Therefore, the objective of this study is to compare the cost-effectiveness of all available CDK4/6 inhibitors (abemaciclib, palbociclib and ribociclib) plus fulvestrant with fulvestrant monotherapy in the second-line treatments for HR+/HER2− advanced or metastatic breast cancer from the perspective of Chinese healthcare system.

## Methods

This economic evaluation was based on the modelling techniques and published literature, and therefore, the ethical approval of the independent ethics committee was exempted because no real human participants or animals were contained. This study followed the consolidated health economic evaluation reporting standards 2022 (Husereau et al., 2022) checklist (Supplementary Table S1).

### Patients and intervention

Eligible patients were women with HR+/HER2− advanced or metastatic breast cancer whose disease had progressed on endocrine therapy, which were divided into four treatment groups: (1) abemaciclib plus fulvestrant group (ABE + FUL): patients received 150 mg abemaciclib twice daily each 28-day cycle plus 500 mg fulvestrant by intramuscular injection on days 1 and 15 of the first cycle and on day 1 of each cycle thereafter; (2) palbociclib plus fulvestrant group (PAL + FUL): patients received 125 mg palbociclib once daily on a 3-week-on, 1-week-off schedule plus 500 mg fulvestrant by intramuscular injection on days 1 and 15 of the first cycle and on day 1 of each cycle thereafter. (3) ribociclib plus fulvestrant group (RIB + FUL): patients received 600 mg ribociclib once daily on a 3-week-on, 1-week-off schedule plus 500 mg fulvestrant by intramuscular injection on days 1 and 15 of the first cycle and on day 1 of each cycle thereafter. (4) fulvestrant monotherapy group: patients only received 500 mg fulvestrant by intramuscular injection on days 1 and 15 of the first cycle and on day 1 of each cycle thereafter. The above-mentioned information was obtained from clinical trials (Cristofanilli et al., 2022; Slamon et al., 2021; Sledge et al., 2020). Treatment continued until disease progression or unacceptable toxicity. After disease progression, best support care was used for these patients as they have already received two lines of therapy.

### Model structure

**FIGURE 1:**
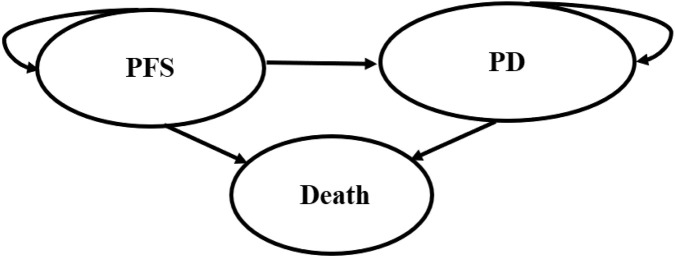
The structure of the partitioned survival model. PFS, progression-free survival; PD, progressive disease.

To compare the cost-effectiveness of four competing regimens, a partitioned survival model was developed with 3 mutually independent health states: progression-free survival (PFS), progressive disease (PD), and death (Figure 1). All patients were set into a PFS state when entering the model, and they could maintain their assigned health state or redistribute to another health state during each cycle. In this model, the proportion of patients in each state over time was directly derived from the PFS and OS curves. The cycle length of the model was set at 28-day with 10-year time horizon. In addition, half-cycle correction was employed to improve the accuracy of the results. This study was conducted from the perspective of Chinese healthcare system. The primary outcomes included the total costs, quality-adjusted life-years (QALYs), and incremental cost-effectiveness ratio (ICER). ICER was described as the incremental cost per quality-adjusted life-year. Costs and QALYs were discounted at an annual rate of 5%, in accordance with the China Guidelines for Pharmacoeconomic Evaluations (Liu et al., 2020). We used 3 times gross domestic product *per capita* ($40,334.05, in 2024) as the willingness-to-pay (WTP) threshold (Liu et al., 2020).

### Clinical data

The clinical efficacy and safety data were derived from the MONARCH 2 (Sledge et al., 2020), PALOMA 3 (Cristofanilli et al., 2022) and MONALEESA 3 (Slamon et al., 2021) trials. To determine the survival data for fulvestrant monotherapy group, the average of the above-mentioned clinical trials was calculated. The PFS and OS curves for patients administered with AEB + FUL, PAL + FUL and RIB + FUL were obtained directly from the MONARCH 2, PALOMA 3 and MONALEESA 3 trials, respectively, because they did not meet the proportion hazard (PH) assumption (Supplementary Figures S1–6). GetData Graph Digitizer 2.26 (http://www.getdata-graph-digitizer.com/) were applied to extract data points from PFS and OS Kaplan-Meier curves, and then reconstructed individual patient data (IPD) over the clinical trial time (Guyot et al., 2012). Five parametric survival model: exponential, Weibull, Log-logistic, Log-normal, and Gompertz, were used to extrapolate the survival curves beyond the follow-up duration of clinical trials (Ishak et al., 2013). The goodness-of-fit was judged based on visual inspection, Akaike information criterion (AIC) and Bayesian information criterion (BIC) (Latimer, 2013). The AIC and BIC were calculated using Stata 15.1. Finally, Log-logistic or Log-normal distributions presented best-fitting for PFS and OS curves, respectively, across three CDK4/6 inhibitors groups. The estimated scale (λ) and shape (γ) parameters of the fitted model are showed in Table 1 (Supplementary Table S2; Supplementary Figures S1–14).

**TABLE 1:** Basic parameters input to the model and the ranges of the sensitivity analysis.

Parameters	Baseline value	Range		Distribution	Reference
		Minimum	Maximum		
Clinical inputs					
PFS: ABE + FUL	shape: 3.026242 scale: −0.287580	NA	NA	Log-logistic	Sledge et al. (2020), Sledge et al. (2017)
OS: ABE + FUL	shape: 3.724941 scale: −0.664670	NA	NA	Log-logistic	Sledge et al. (2020)
PFS: PAL + FUL	shape: 2.404594 scale: −0.380963	NA	NA	Log-logistic	Cristofanilli et al. (2022)
OS: PAL + FUL	shape: 3.553685 scale: −0.143696	NA	NA	Log-normal	Cristofanilli et al. (2022)
PFS: RIB + FUL	shape: 2.591883 scale: 0.129698	NA	NA	Log-normal	Slamon et al. (2021)
OS: RIB + FUL	shape: 3.773049 scale: −0.023510	NA	NA	Log-normal	Slamon et al. (2021)
PFS: FUL-MONARCH2	shape: 0.051149 scale: 0.0352083	NA	NA	Gompertz	Sledge et al. (2020), Sledge et al. (2017)
OS: FUL-MONARCH2	shape: 0.0063987 scale: 0.0343997	NA	NA	Gompertz	Sledge et al. (2020)
PFS: FUL-PALOMA3	shape: 1.901407 scale: 0.172908	NA	NA	Log-normal	Cristofanilli et al. (2022)
OS: FUL-PALOMA3	shape: 3.449401 scale: −0.699308	NA	NA	Log-logistic	Cristofanilli et al. (2022)
PFS: FUL-MONALEESA3	shape: 2.236723 scale: 0.176998	NA	NA	Log-normal	Slamon et al. (2021)
OS: FUL-MONALEESA3	shape: 0.005013 scale: 0.334347	NA	NA	Weibull	Slamon et al. (2021)
Cost inputs (US $)					
abemaciclib (1 mg)	0.07	0.06	0.09	Gamma	YaoZH (2025)
palbociclib (1 mg)	0.11	0.09	0.14	Gamma	YaoZH (2025)
ribociclib (1 mg)	0.05	0.04	0.06	Gamma	YaoZH (2025)
fulvestrant (1 mg)	0.36	0.28	0.43	Gamma	YaoZH (2025)
drug administration	33.56	26.85	40.27	Gamma	Bao et al. (2022)
imaging tests	176.49	141.19	211.79	Gamma	Zeng et al. (2023)
laboratory tests	82.59	66.07	99.11	Gamma	Zeng et al. (2023)
best supportive care	2155.71	1724.57	2586.85	Gamma	Tang et al. (2022)
terminal care in end-of-life	1460.30	1055.30	2085.70	Gamma	Wu et al. (2014)
management cost of diarrhea	38.87	31.09	46.64	Gamma	Shi et al. (2018)
management cost of neutropenia	461.50	369.20	553.80	Gamma	Wu et al. (2012)
management cost of anemia	531.70	425.36	638.04	Gamma	Wu et al. (2012)
management cost of leukopenia	435.58	348.46	522.70	Gamma	Zhu et al. (2023)
management cost of infections	395.82	316.66	474.98	Gamma	Zhu et al. (2023)
management cost of hepatobiliary toxicity	87.30	69.84	104.76	Gamma	Wen et al. (2021)
Utility inputs					
PFS	0.84	0.67	1.00	Beta	Mistry et al. (2018)
PD	0.44	0.35	0.53	Beta	Lloyd et al. (2006)
Disutility inputs					
diarrhea	0.10	0.08	0.12	Beta	Lloyd et al. (2006)
neutropenia	0.15	0.12	0.18	Beta	Lloyd et al. (2006)
anemia	0.12	0.10	0.14	Beta	Zhu et al. (2022)
leukopenia	0.15	0.12	0.18	Beta	Assumption
infections	0.15	0.12	0.18	Beta	Huang et al. (2021)
hepatobiliary toxicity	0.10	0.08	0.12	Beta	Huang et al. (2021)
Risk of severe adverse events in ABE + FUL					
diarrhea	0.15	0.12	0.17	Beta	Sledge et al. (2020)
neutropenia	0.30	0.24	0.36	Beta	Sledge et al. (2020)
anemia	0.09	0.07	0.11	Beta	Sledge et al. (2020)
leukopenia	0.11	0.09	0.13	Beta	Sledge et al. (2020)
Risk of severe adverse events in PAL + FUL					
neutropenia	0.70	0.56	0.84	Beta	Cristofanilli et al. (2022)
leukopenia	0.39	0.31	0.46	Beta	Cristofanilli et al. (2022)
infections	0.06	0.05	0.07	Beta	Cristofanilli et al. (2022)
Risk of severe adverse events in RIB + FUL					
neutropenia	0.58	0.47	0.70	Beta	Slamon et al. (2021)
leukopenia	0.17	0.14	0.20	Beta	Slamon et al. (2021)
infections	0.08	0.06	0.10	Beta	Slamon et al. (2021)
hepatobiliary toxicity	0.14	0.11	0.17	Beta	Slamon et al. (2021)
Risk of severe adverse events in FUL					
hepatobiliary toxicity	0.06	0.05	0.07	Beta	Slamon et al. (2021)
Subsequent therapy proportion					
ABE + FUL	0.63	0.50	0.76	Beta	Sledge et al. (2020)
PAL + FUL	0.77	0.62	0.92	Beta	Cristofanilli et al. (2022)
RIB + FUL	0.82	0.66	0.98	Beta	Slamon et al. (2021)
FUL	0.83	0.67	1.00	Beta	Cristofanilli et al. (2022), Slamon et al. (2021), Sledge et al. (2020)
Others					
discount rate	0.05	0.00	0.08	Beta	Liu et al. (2020)

ABE, abemaciclib; PAL, palbociclib; RIB, ribociclib; FUL, fulvestrant; PFS, progression-free survival; OS, overall survival.

### Costs

Only direct medical costs were considered, including drug acquisition costs, drug administration costs, follow up costs (including imaging and laboratory tests), best supportive care costs, management costs associated with severe adverse events (AEs), and terminal care costs in end-of-life. The drug dose, duration of treatment, proportion of subsequent therapies, and incidence of AEs for different regimes were in line with the clinical trials. Only grade 3 or 4 AEs with a frequency ≥5% were considered to simplify the model. The unit price of drugs was retrieved from local database in China (YaoZH, 2025). Other relevant costs were derived from previously published literature (Bao et al., 2022; Shi et al., 2018; Tang et al., 2022; Wen et al., 2021; Wu et al., 2012; Wu et al., 2014; Zeng et al., 2023; Zhu et al., 2023). All cost was converted into US dollars (1$ = 7.12 CNY), and adjusted based on the Consumer Price Index for inflation to reflect 2024 US dollars.

### Utilities

Each health state was assigned a utility value on a scale of 0 (death) to 1 (perfect health). The utility values of PFS state were derived from an economic evaluation in which the data were collected from the MONALEESA 2 trial, measured using the EuroQol five dimensions health status questionnaire (EQ-5D-5L) and calculated with UK-specific value algorithm (Mistry et al., 2018). Utility value for PD state was derived from published study (Lloyd et al., 2006), which estimated utility values employing standard gambling techniques. The disutility values caused by grade 3 or 4 treatment-related AEs were taken from published sources by multiplying the duration-adjusted disutilities by the prevalence rates of specific AEs (Huang et al., 2021; Lloyd et al., 2006; Zhu et al., 2022). The details of costs and utilities are presented in Table 1.

### Sensitivity analysis

The robustness of the base-case results was evaluated by one-way and probabilistic sensitivity analyses (PSA). One-way sensitivity analyses were performed to assess the effect of input parameters on ICER by adjusting the parameters to a plausible range (±20%) or 95% confidence intervals. The results displayed using Tornado diagrams. PSA was conducted based on Monte Carlo simulation with 10,000 iterations by simultaneously sampling the key parameters from defined statistical distributions. Gamma distributions were adopted for costs, and beta distributions were selected for probabilities of AEs, proportion of subsequent treatment, utilities and discount rate. The result of PSA was showed as cost-effectiveness acceptability curves to illustrate the probability of ABE + FUL, PAL + FUL, RIB + FUL and FUL would be considered optimal treatment at various WTP thresholds. In addition, a lifetime horizon was used to calculate ICERs to ensure that all patients were entered into death states to explore the impact of time horizon on the base-case results.

## Results

### Base-case results

The base-case results are shown in Table 2. Compared with the FUL, ABE + FUL, PAL + FUL and RIB + FUL as the second-line therapy for HR+/HER2− advanced or metastatic breast cancer provided incremental costs of $2,767.88, $298.77, $22,290.47, with additional QALYs of 0.76, 0.24, 0.56, respectively, resulting in ICERs of $3,636.51/QALY, $1,256.32/QALY and $39,654.78/QALY. Notably, in the pairwise comparison between three competing CDK4/6 inhibitors, ABE + FUL represented the optimal option for HR+/HER2− advanced or metastatic breast cancer patients at the current price and WTP threshold.

**TABLE 2:** Base case results.

Strategy	Total costs ($)	Total QALYs	ICER ($/QALY, pairwise comparison)^a^		
FUL	57,857.97	1.70	FUL		
PAL + FUL	58,156.74	1.94	1,256.32	PAL + FUL	
ABE + FUL	60,625.85	2.46	3,636.51	4,718.12	ABE + FUL
RIB + FUL	80,148.45	2.26	39,654.78	67,812.36	Dominated

ABE, abemaciclib; PAL, palbociclib; RIB, ribociclib; FUL, fulvestrant; QALYs, quality-adjusted life years; ICER, incremental cost-effectiveness ratio.

^a^ Other treatment regimes compared with treatment options in the first row.

### Sensitivity analysis

**FIGURE 2:**
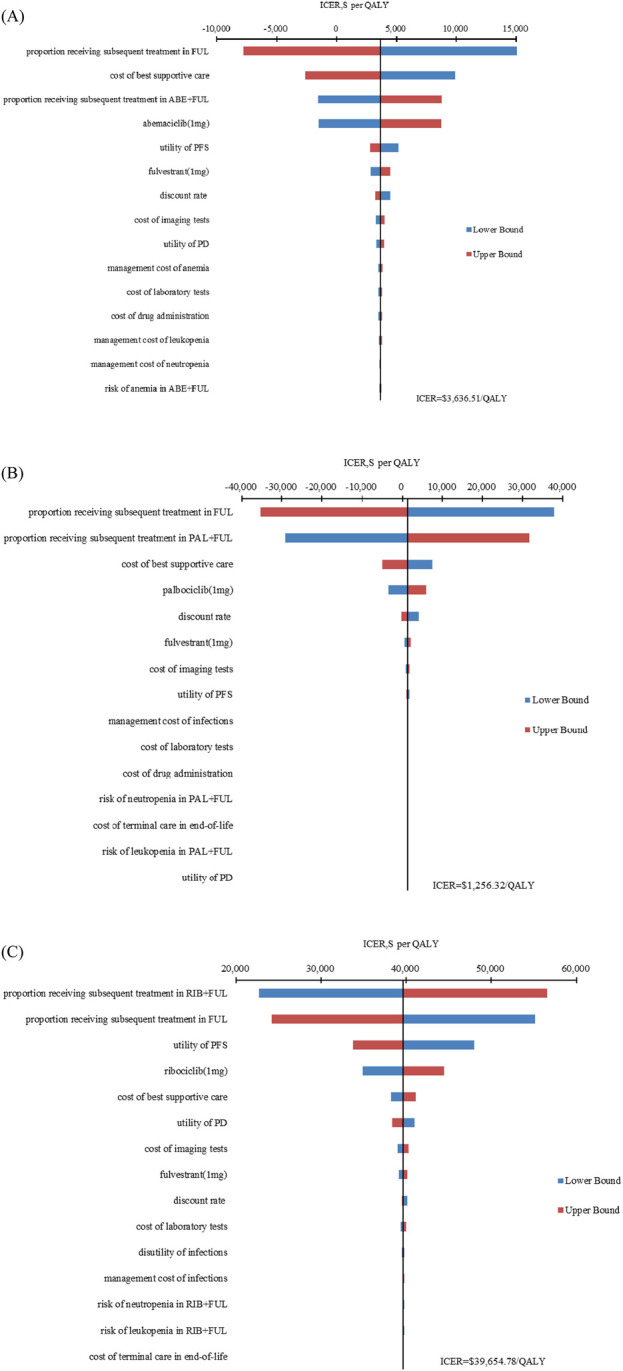
Tornado diagram of one-way sensitivity analysis. **(A)** ABE + FUL versus FUL; **(B)** PAL + FUL versus FUL; **(C)** RIB + FUL versus FUL. ABE, abemaciclib; PAL, palbociclib; RIB, ribociclib; FUL, fulvestrant; ICER, incremental cost-effectiveness ratio; QALY, quality-adjusted life year; PFS, progression-free survival; PD, progressive disease.

**FIGURE 3:**
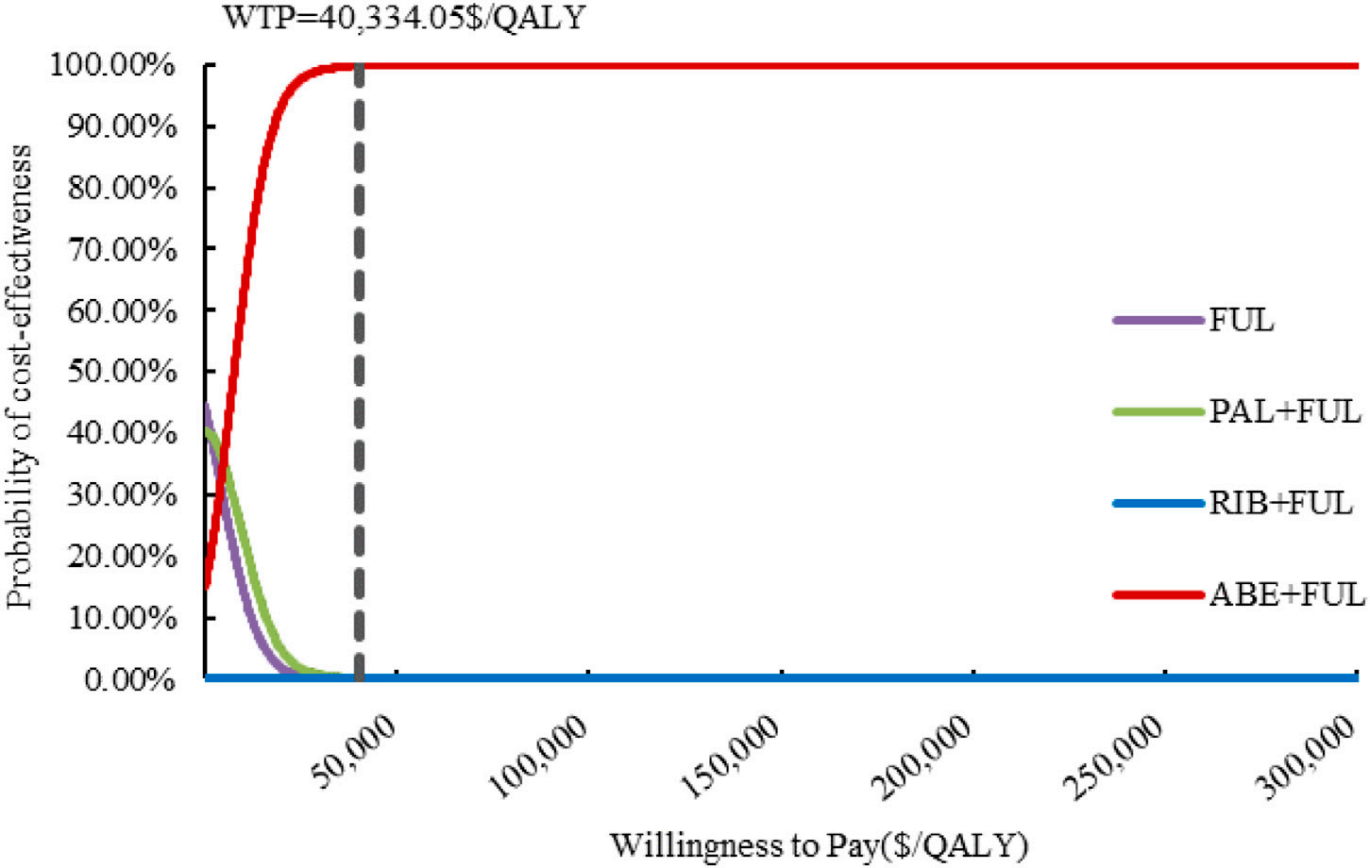
Cost-effectiveness acceptability curves. ABE, abemaciclib; PAL, palbociclib; RIB, ribociclib; FUL, fulvestrant; QALY, quality-adjusted life year; WTP, willingness to pay.

The results of one-way sensitivity analysis are presented in Figure 2. The proportion of patients received subsequent treatment, utility values of PFS, cost of best supportive care played a considerable role in base-case results. Compared with FUL, the ICERs of ABE + FUL and PAL + FUL were consistently lower than the WTP threshold regardless of model parameter variations, which validated the robustness of our model. For RIB + FUL vs. FUL, alterations in the proportion of patients received subsequent treatment, utility values of PFS and PD, the price of ribociclib, and cost of best supportive care could significantly alter the conclusion. At the current price and WTP threshold, ABE + FUL achieved an overwhelming superiority with a 99.82% probability to be the optimal option in China (Figure 3). At the lifetime horizon, the findings were not substantially altered, namely, ABE + FUL was the most cost-effective option against competing regimens (Supplementary Table S3).

## Discussion

In recent years, CDK4/6 inhibitors have been extensively used in second-line treatment for HR+/HER2− advanced or metastatic breast cancer. The available network meta-analyses revealed that ABE + FUL provided the optimal OS (Shao et al., 2024); however, the most cost-effective option remained unclear in China. Thus, we incorporated the latest clinical evidence to investigate the cost-effectiveness of all available CDK4/6 inhibitors as the second-line treatment for HR+/HER2− advanced or metastatic breast cancer in China. The results revealed that abemaciclib, palbociclib and ribociclib combined with fulvestrant were cost-effective compared to fulvestrant monotherapy. Abemaciclib plus fulvestrant was the most cost-effective treatment paradigm under the current WTP threshold.

The findings could help to choose individualized treatments and benefit decision-makers in making optimal decisions. In the PFS state, ABE + FUL could accumulate more QALYs than palbociclib or ribociclib plus fulvestrant. In the PD state, ABE + FUL produced much lower QALYs than palbociclib or ribociclib plus fulvestrant, but the concomitant cost of treatment was lower, which benefited from the longer PFS and similar OS of ABE + FUL. As a result, ABE + FUL became the cost-effective treatment strategy. Moreover, the interim results of the DAWNA-1 (Xu et al., 2021), a phase III trial, confirmed that dalpiciclib plus fulvestrant significantly improved the PFS compared to placebo plus fulvestrant (15.7 vs. 7.2 months, hazard ratio [HR], 0.42; 95% confidence interval [CI], 0.31 to 0.58, P < 0.001). Therefore, further analyses will be conducted in conjunction with dalpiciclib plus fulvestrant when the final data are published.

From the results of medical insurance negotiations announced by National Healthcare Security Administration (NHSA) in 2024, the adjustment added 91 new drugs, with an average price reduction of 63%, including 26 anticancer drugs (Central People’s Government of the People’s Republic of China, 2024). Our results found that RIB + FUL is not cost-effective in China at current price when a lifelong time horizon was adapted, with a WTP threshold of $40,334.05/QALY. At present, without further breakthroughs in efficacy, substantial price reductions are crucial to ensuring the cost-effectiveness and affordability of treatment regimens, especially in countries with heavy cancer burdens and limited healthcare resources (Liu et al., 2022; Wu et al., 2022). Huang et al. evaluated the cost-effectiveness of ribociclib as the first-line treatment for premenopausal women with HR+/HER2− advanced breast cancers from the perspectives of the Chinese healthcare system and determined the potential price for ribociclib in China based on its price in the United States, concluding that if the cost of ribociclib was below $31.74/200 mg, the probability of cost-effectiveness approached 50% (Huang et al., 2021). Besides, patient assistance program (PAP) is often launched in China, which provided free medication for low-income or poor cancer patients who meet the medical or financial criteria of the program (Zhu et al., 2023).

To date, several published studies have evaluated the cost-effectiveness of CDK4/6 inhibitors in the second-line treatments for HR+/HER2− advanced or metastatic breast cancer. Zhang et al. (2019) and Zhu et al. (2023) estimated the cost-effectiveness of PAL + FUL based on PALOMA 3 trial, respectively, and suggested that PAL + FUL was unlikely to be cost-effective in comparison with fulvestrant in China. As the medical insurance negotiation mechanism has dramatically improved accessibility for patients, the prices used in previous assessment were no longer currently applicable. Our results showed that PAL + FUL was a cost-effective regime compared with fulvestrant monotherapy. Wang et al. (2021) performed an economic evaluation of ABE + FUL versus PAL + FUL, RIB + FUL, and fulvestrant monotherapy in the second-line treatment for HR+/HER2− advanced or metastatic breast cancer from the US payer perspective, and demonstrated that fulvestrant monotherapy would be the most cost-effective treatment among these four options under the US WTP threshold. As for three CDK4/6 inhibitors, PAL + FUL had the highest probability to be cost-effective. Colombo et al. (2023) compared three CDK4/6 inhibitors in association with fulvestrant in the context of Italian healthcare system, the results indicated that PAL + FUL produced slight cost savings over a lifetime horizon. Our findings were not consistent with foreign economic evaluations, which might be attributed to structural differences in drug pricing and reimbursement frameworks across countries, rather than clinically meaningful differences in efficacy or safety.

There are several limitations should be considered. First, in the absence of direct head-to-head comparison, there might be heterogeneity in the comparation between various treatment options. However, we used clinical evidence from three clinical trials, MONARCH 2, PALOMA 3 and MONALEESA 3 trials, in which the baseline characteristics of participants were highly similar, making the heterogeneity to be limited. Second, several key cost parameters, such as test, best supportive care and terminal care, were obtained from previously published literature rather than more representative and appropriate real-world data. But the sensitivity analysis indicated that these variables had little effect on the base-case results. Third, the management cost of grade 1-2 AEs with incidence below 5% were not included in the analysis. Fortunately, one-way sensitivity analyses found that the model results were insensitive to AEs-related inputs. Fourth, as subsequent treatments were not defined in the clinical trials, we hypothesized that BSC was the primary subsequent option in consonance with clinical guideline, which might be distinct from the realistic medication choices.

## Conclusion

In summary, compared with fulvestrant monotherapy, abemaciclib plus fulvestrant, palbociclib plus fulvestrant and ribociclib plus fulvestrant were cost-effectiveness in the second-line treatment for patients with HR+/HER2− advanced or metastatic breast cancer from the perspective of Chinese healthcare system. In the pairwise comparison between available CDK4/6 inhibitors, abemaciclib plus fulvestrant was optimal option in China.

## Data Availability

The raw data supporting the conclusions of this article will be made available by the authors, without undue reservation.
