# Supplementary material for: Cost-effectiveness analysis of CDK4/6 inhibitors in the second-line treatment for HR+/HER2− advanced or metastatic breast cancer
Source: Front Pharmacol. 2025 Sep 8;16:1540088. doi: 10.3389/fphar.2025.1540088 (PMC12450880; doi:10.3389/fphar.2025.1540088)
Supplement: Supplementary file 1 [file Supplementaryfile1.doc]

**Cost-effectiveness analysis of CDK4/6 inhibitors in the second-line treatment for HR+/HER2- advanced or metastatic breast cancer**

Kaixuan Wang^1†^, Shixian Liu^2†^, Shunping Li^2^ and Jie Gao^1*^

^1^Shandong Provincial Maternal and Child Health Care Hospital Affiliated to Qingdao University, Jinan, China

^2^Centre for Health Management and Policy Research, School of Public Health, Cheeloo College of Medicine, Shandong University, Jinan, China

^†^These authors have contributed equally to this work..

^*^Correspondence: Jie Gao

agao1224@163.com

**Supplementary Materials**

**Supplementary Table 1**. CHEERS Checklist 2022

**Supplementary Table 2**. Summary of statistical goodness-of-fit of Kaplan-Meier curves

**Supplementary Table 3**. Scenario analysis results

**Supplementary Figure 1**. The test of PH assumption of OS of abemaciclib plus fulvestrant

**Supplementary Figure 2**. The test of PH assumption of PFS of abemaciclib plus fulvestrant

**Supplementary Figure 3**. The test of PH assumption of OS of palbociclib plus fulvestrant

**Supplementary Figure 4**. The test of PH assumption of PFS of palbociclib plus fulvestrant

**Supplementary Figure 5**. The test of PH assumption of OS of ribociclib plus fulvestrant

**Supplementary Figure 6**. The test of PH assumption of PFS of ribociclib plus fulvestrant

**Supplementary Figure 7**. The reconstructed Kaplan-Meier PFS curves of fulvestrant monotherapy

**Supplementary Figure 8**. The reconstructed Kaplan-Meier OS curves of fulvestrant monotherapy

**Supplementary Figure 9**. The reconstructed Kaplan-Meier PFS curves of abemaciclib plus fulvestrant

**Supplementary Figure 10**. The reconstructed Kaplan-Meier OS curves of abemaciclib plus fulvestrant

**Supplementary Figure 11**. The reconstructed Kaplan-Meier PFS curves of palbociclib plus fulvestrant

**Supplementary Figure 12**. The reconstructed Kaplan-Meier OS curves of palbociclib plus fulvestrant

**Supplementary Figure 13**. The reconstructed Kaplan-Meier PFS curves of ribociclib plus fulvestrant

**Supplementary Figure 14**. The reconstructed Kaplan-Meier OS curves of ribociclib plus fulvestrant

**Supplementary Table 1**. CHEERS Checklist 2022

| Section | Item  No | Guidance for reporting | Reported |
| --- | --- | --- | --- |
| **Title** | | | |
| Title | 1 | Identify the study as an economic evaluation and specify the interventions being compared | Yes |
| **Abstract** | | | |
| Abstract | 2 | Provide a structured summary that highlights context, key  methods, results, and alternative analyses | Yes |
| **Introduction** | | | |
| Background and objectives | 3 | Give the context for the study, the study question, and its practical relevance for decision making in policy or practice | Yes |
| **Methods** | | | |
| Health economic analysis plan | 4 | Indicate whether a health economic analysis plan was developed and where available | Yes |
| Study population | 5 | Describe characteristics of the study population (such as age range, demographics, socioeconomic, or clinical characteristics) | Yes |
| Setting and location | 6 | Provide relevant contextual information that may influence findings | Yes |
| Comparators | 7 | Describe the interventions or strategies being compared and why chosen | Yes |
| Perspective | 8 | State the perspective(s) adopted by the study and why chosen | Yes |
| Time horizon | 9 | State the time horizon for the study and why appropriate | Yes |
| Discount rate | 10 | Report the discount rate(s) and reason chosen | Yes |
| Selection of outcomes | 11 | Describe what outcomes were used as the measure(s) of benefit(s) and harm(s) | Yes |
| Measurement of outcomes | 12 | Describe how outcomes used to capture benefit(s) and harm(s) were measured | Yes |
| Valuation of outcomes | 13 | Describe the population and methods used to measure and value outcomes | Yes |
| Measurement and valuation of resources and costs | 14 | Describe how costs were valued | Yes |
| Currency, price date, and conversion | 15 | Report the dates of the estimated resource quantities and unit costs, plus the currency and year of conversion | Yes |
| Rationale and description of model | 16 | If modelling is used, describe in detail and why used. Report if the model is publicly available and where it can be accessed | Yes |
| Analytics and assumptions | 17 | Describe any methods for analysing or statistically transforming data, any extrapolation methods, and approaches for validating  any model used | Yes |
| Characterising heterogeneity | 18 | Describe any methods used for estimating how the results of the study vary for subgroups | Yes |
| Characterising distributional effects | 19 | Describe how impacts are distributed across different individuals or adjustments made to reflect priority populations | Yes |
| Characterising uncertainty | 20 | Describe methods to characterise any sources of uncertainty in the analysis | Yes |
| Approach to engagement with patients and others affected by the study | 21 | Describe any approaches to engage patients or service recipients, the general public, communities, or stakeholders (such as clinicians or payers) in the design of the study | Yes |
| **Results** | | | |
| Study parameters | 22 | Report all analytic inputs (such as values, ranges, references) including uncertainty or distributional assumptions | Yes |
| Summary of main results | 23 | Report the mean values for the main categories of costs and outcomes of interest and summarise them in the most appropriate overall measure | Yes |
| Effect of uncertainty | 24 | Describe how uncertainty about analytic judgments, inputs, or projections affect findings. Report the effect of choice of discount rate and time horizon, if applicable | Yes |
| Effect of engagement with patients  and others affected by the study | 25 | Report on any difference patient/service recipient, general  public, community, or stakeholder involvement made to the approach or findings of the study | Yes |
| **Discussion** | | | |
| Study findings, limitations, generalisability, and current knowledge | 26 | Report key findings, limitations, ethical or equity considerations not captured, and how these could affect patients, policy, or practice | Yes |
| Other relevant information Source of funding | 27 | Describe how the study was funded and any role of the funder in the identification, design, conduct, and reporting of the analysis | Yes |
| Conflicts of interest | 28 | Report authors conflicts of interest according to journal or  International Committee of Medical Journal Editors requirements | Yes |

**Supplementary Table 2**. Summary of statistical goodness-of-fit of Kaplan-Meier curves

|  | Exponential | Weibull | Log-logistic | Log-normal | Gompertz |
| --- | --- | --- | --- | --- | --- |
| **ABE+FUL-PFS** | | | | | |
| parameter 1 | 0.032362 | 0.024395 | 3.026242 | 3.015934 | 0.031242 |
| parameter 2 | - | 0.085882 | -0.287580 | 0.240305 | 0.002781 |
| AIC | 1213.519000 | 1213.042000 | 1207.758000^*^ | 1197.217000^#^ | 1215.365000 |
| BIC | 1217.620000 | 1221.242000 | 1215.959000^*^ | 1205.418000^#^ | 1223.566000 |
| **ABE+FUL-OS** | | | | | |
| parameter 1 | 0.017400 | 0.001970 | 3.724941 | 3.701317 | 0.008501 |
| parameter 2 | - | 0.444964 | -0.664670 | -0.073184 | 0.024900 |
| AIC | 1121.908000 | 1055.850000^#^ | 1067.145000^*^ | 1081.466000 | 1064.141000 |
| BIC | 1126.008000 | 1064.051000^#^ | 1075.346000^*^ | 1089.666000 | 1072.342000 |
| **PAL+FUL-PFS** | | | | | |
| parameter 1 | 0.059369 | 0.043470 | 2.404594 | 2.386617 | 0.058168 |
| parameter 2 | - | 0.105391 | -0.380963 | 0.139850 | 0.002228 |
| AIC | 1006.788000 | 1004.985000 | 996.249700^*^ | 987.387400^#^ | 1008.731000 |
| BIC | 1010.638000 | 1012.683000 | 1003.948000^*^ | 995.086000^#^ | 1016.430000 |
| **PAL+FUL-OS** | | | | | |
| parameter 1 | 0.020372 | 0.004604 | 3.559452 | 3.553685 | 0.015539 |
| parameter 2 | - | 0.316455 | -0.677187 | -0.143696 | 0.008578 |
| AIC | 934.685200 | 898.537400 | 876.272100 | 871.384800^#*^ | 924.733600 |
| BIC | 938.534500 | 906.236000 | 883.970700 | 879.083400^#*^ | 932.432300 |
| **RIB+FUL-PFS** | | | | | |
| parameter 1 | 0.046180 | 0.038927 | 2.619276 | 2.591883 | 0.048142 |
| parameter 2 | - | 0.050738 | -0.389341 | 0.129698 | -0.002697 |
| AIC | 725.636300 | 726.856200 | 722.581100 | 714.206700^#*^ | 727.406300 |
| BIC | 729.104400 | 733.792300 | 729.517200 | 721.142800^#*^ | 734.342400 |
| **RIB+FUL-OS** | | | | | |
| parameter 1 | 0.015680 | 0.003712 | 3.770195 | 3.773049 | 0.012022 |
| parameter 2 | - | 0.311248 | -0.576657 | -0.023510 | 0.009210 |
| AIC | 612.911900 | 597.070600 | 586.082000 | 586.023700^#*^ | 609.833400 |
| BIC | 616.379900 | 604.006700 | 593.018100 | 592.959900^#*^ | 616.769500 |
| **FUL-MONARCH2-PFS** | | | | | |
| parameter 1 | 0.075415 | 0.038075 | 2.227266 | 2.184016 | 0.051149 |
| parameter 2 | - | 0.212449 | -0.510961 | -0.013874 | 0.035208 |
| AIC | 631.569700 | 620.255400 | 644.425200 | 626.840400 | 616.903100^#*^ |
| BIC | 634.976900 | 627.069800 | 651.239600 | 633.654800 | 623.717500^#*^ |
| **FUL-MONARCH2-OS** | | | | | |
| parameter 1 | 0.017792 | 0.001140 | 3.743107 | 3.699955 | 0.006399 |
| parameter 2 | - | 0.534111 | -0.728130 | -0.105586 | 0.034400 |
| AIC | 554.414300 | 507.951900 | 524.524700 | 537.095700 | 501.459300^#*^ |
| BIC | 557.821500 | 514.766300 | 531.339000 | 543.910100 | 508.273600^#*^ |
| **FUL-PALOMA3-PFS** | | | | | |
| parameter 1 | 0.086823 | 0.110127 | 1.820385 | 1.901407 | 0.138210 |
| parameter 2 | - | -0.098098 | -0.313538 | 0.172908 | -0.069061 |
| AIC | 558.150800 | 558.087300 | 527.580900 | 519.723200^#*^ | 539.609600 |
| BIC | 561.309900 | 564.405400 | 533.899100 | 526.041300^#*^ | 545.927700 |
| **FUL-PALOMA3-OS** | | | | | |
| parameter 1 | 0.023115 | 0.005205 | 3.449401 | 3.435968 | 0.017225 |
| parameter 2 | - | 0.323071 | -0.699308 | -0.143930 | 0.010026 |
| AIC | 469.047500 | 450.186700 | 440.703800^#*^ | 441.594000 | 463.506800 |
| BIC | 472.206500 | 456.504800 | 447.022000^#*^ | 447.912100 | 469.824900 |
| **FUL-MONALEESA3-PFS** | | | | | |
| parameter 1 | 0.060390 | 0.077128 | 2.216332 | 2.236723 | 0.0832700 |
| parameter 2 | - | -0.082637 | -0.336284 | 0.176998 | -0.025769 |
| AIC | 358.012600 | 358.925900 | 346.123400 | 342.270700^#*^ | 352.199100 |
| BIC | 360.704000 | 364.308600 | 351.506100 | 347.653400^#*^ | 357.581800 |
| **FUL-MONALEESA3-OS** | | | | | |
| parameter 1 | 0.022973 | 0.005013 | 3.470459 | 3.423146 | 0.014475 |
| parameter 2 | - | 0.334347 | -0.621266 | -0.037566 | 0.016898 |
| AIC | 298.450000 | 288.005300^#*^ | 291.610400 | 294.639000 | 291.250300 |
| BIC | 301.150500 | 293.406200^#*^ | 297.011300 | 300.040000 | 296.651300 |
| * best fitted model; # best visual inspection; AIC, Akaike information criterion; BIC, Bayesian Information Criterion; ABE, abemaciclib; PAL, palbociclib; RIB, ribociclib; FUL, fulvestrant; PFS, progression-free survival; OS, overall survival. | | | | | |

**Supplementary Table 3**. Scenario analysis results

| **Strategy** | **Total Costs ($)** | **Total QALYs** | **ICER($/QALY, pairwise comparison)^*^** | | |
| --- | --- | --- | --- | --- | --- |
| **Lifetime horizon** | | | | | |
| FUL | 57,900.46 | 1.70 | FUL |  |  |
| PAL+FUL | 60,449.49 | 2.02 | 7,991.64 | PAL+FUL |  |
| ABE+FUL | 64,149.04 | 2.64 | 6,676.10 | 5,996.02 | ABE+FUL |
| RIB+FUL | 87,545.33 | 2.41 | 41,689.74 | 69,100.76 | Dominated |
| ABE, abemaciclib; PAL, palbociclib; RIB, ribociclib; FUL fulvestrant; QALYs quality-adjusted life years; ICER incremental cost-effectiveness ratio.  *Other treatment regimes compared with treatment options in the first row | | | | | |


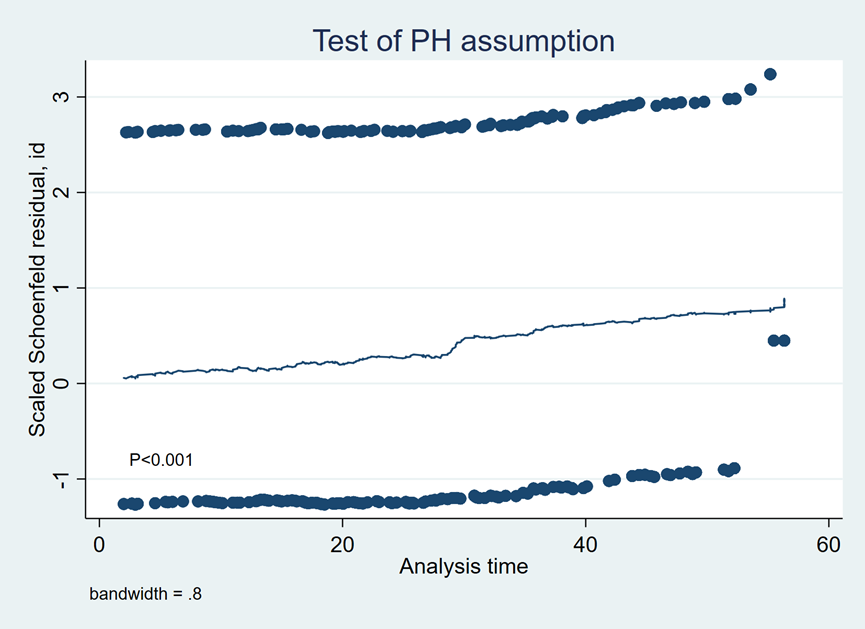

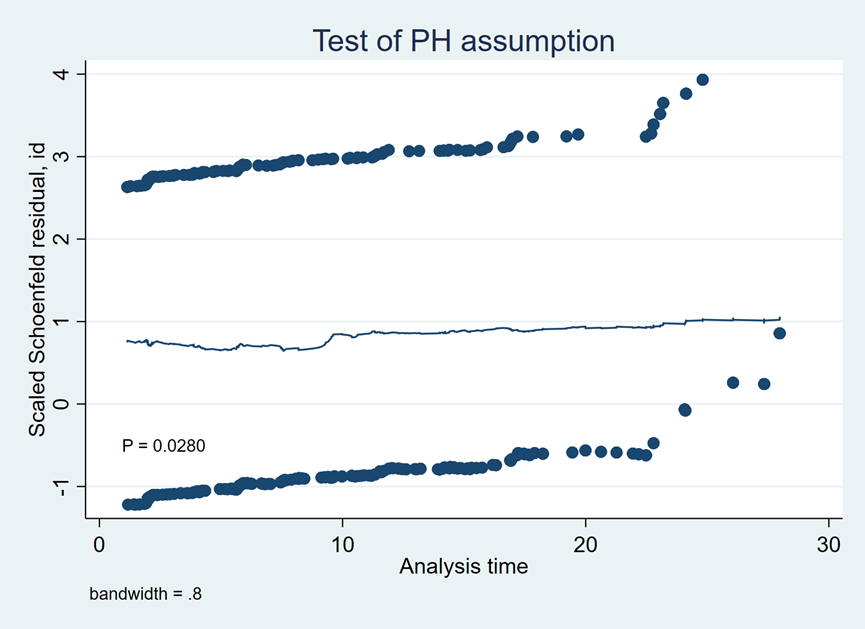
**Supplementary Figure 1**. The test of PH assumption of OS of abemaciclib plus fulvestrant

**Supplementary Figure 2**. The test of PH assumption of PFS of abemaciclib plus fulvestrant


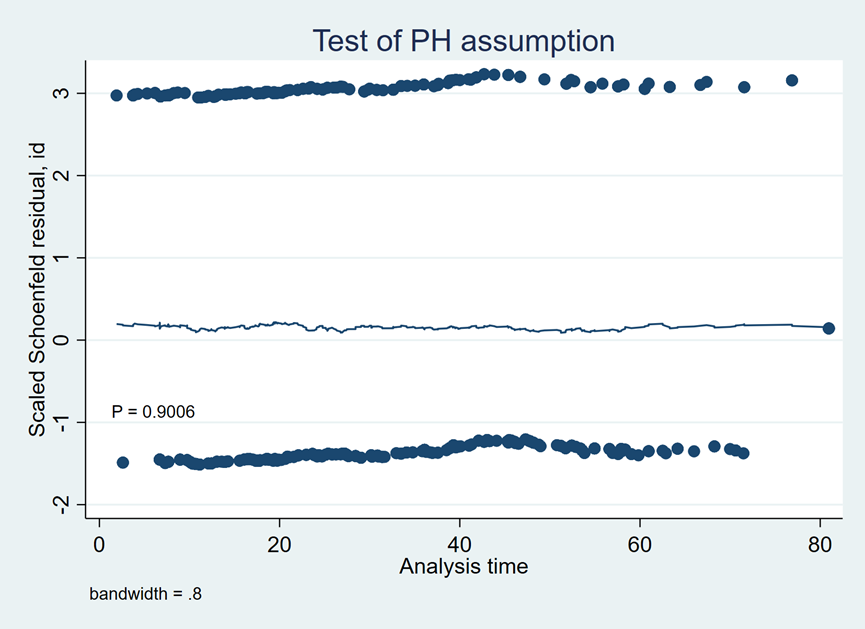


**Supplementary Figure 3**. The test of PH assumption of OS of palbociclib plus fulvestrant


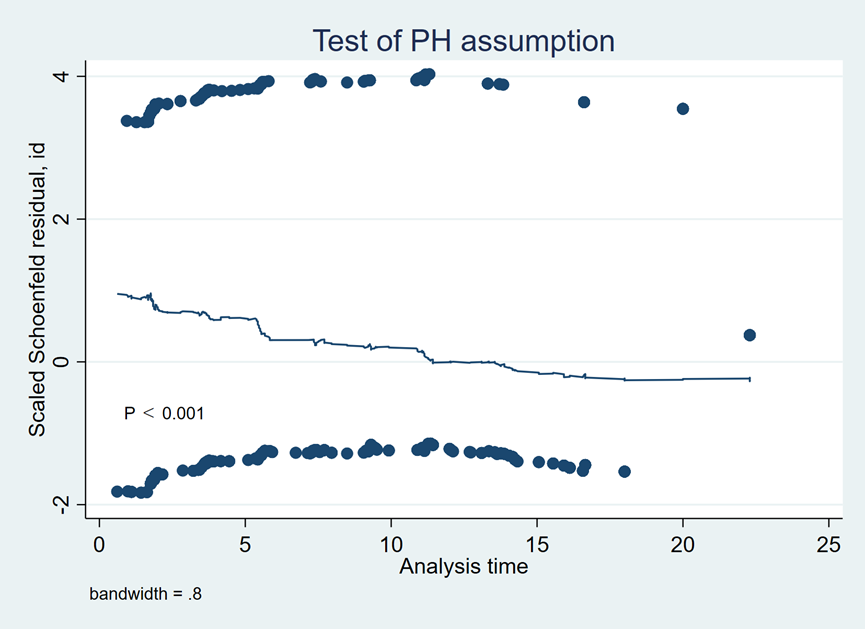


**Supplementary Figure 4**. The test of PH assumption of PFS of palbociclib plus fulvestrant


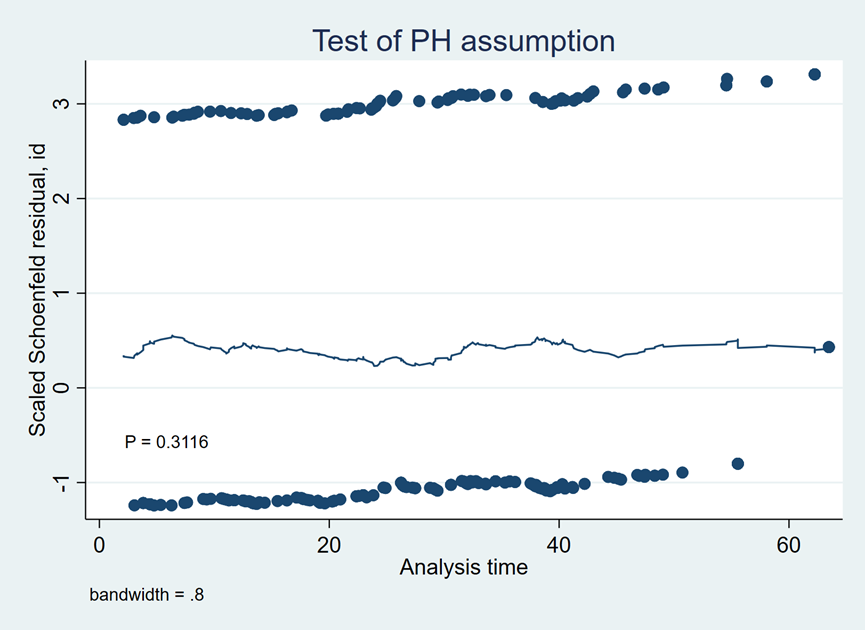

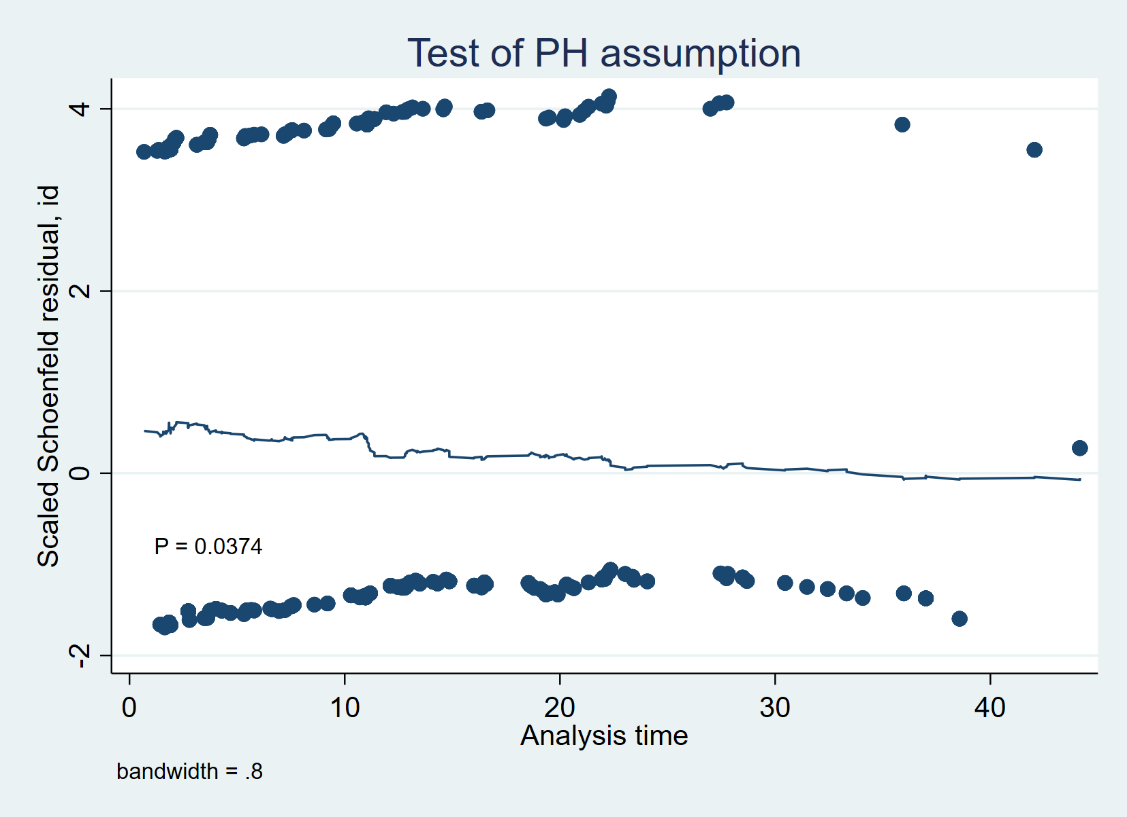
**Supplementary Figure 5**. The test of PH assumption of OS of ribociclib plus fulvestrant

**Supplementary Figure 6**. The test of PH assumption of PFS of ribociclib plus fulvestrant


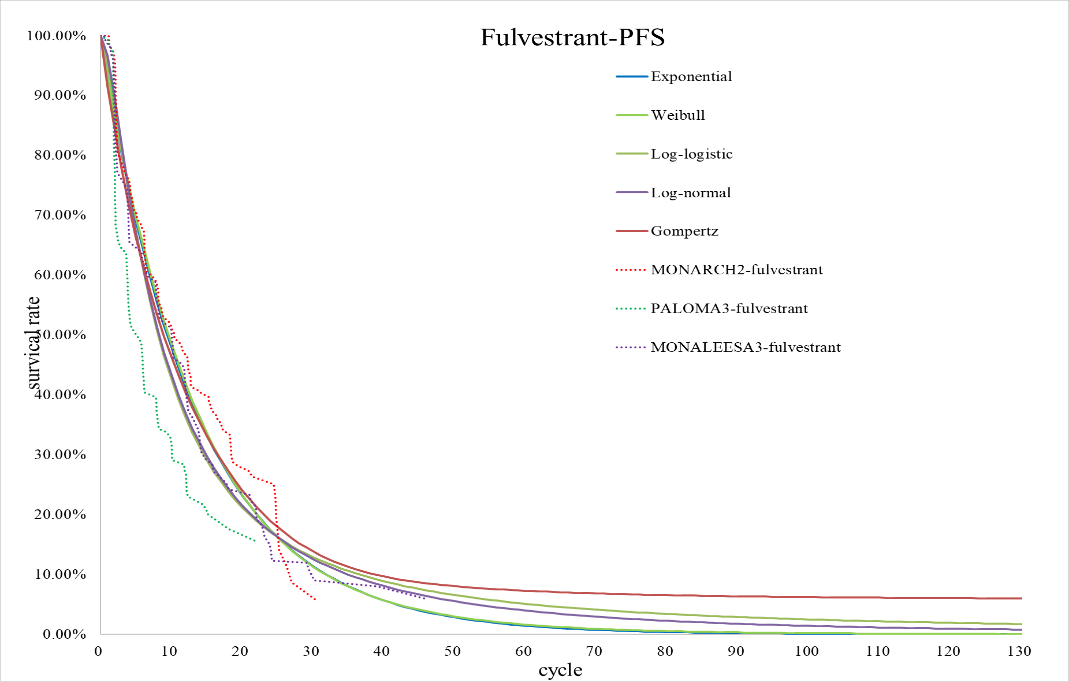


**Supplementary Figure 7**. The reconstructed Kaplan-Meier PFS curves of fulvestrant monotherapy


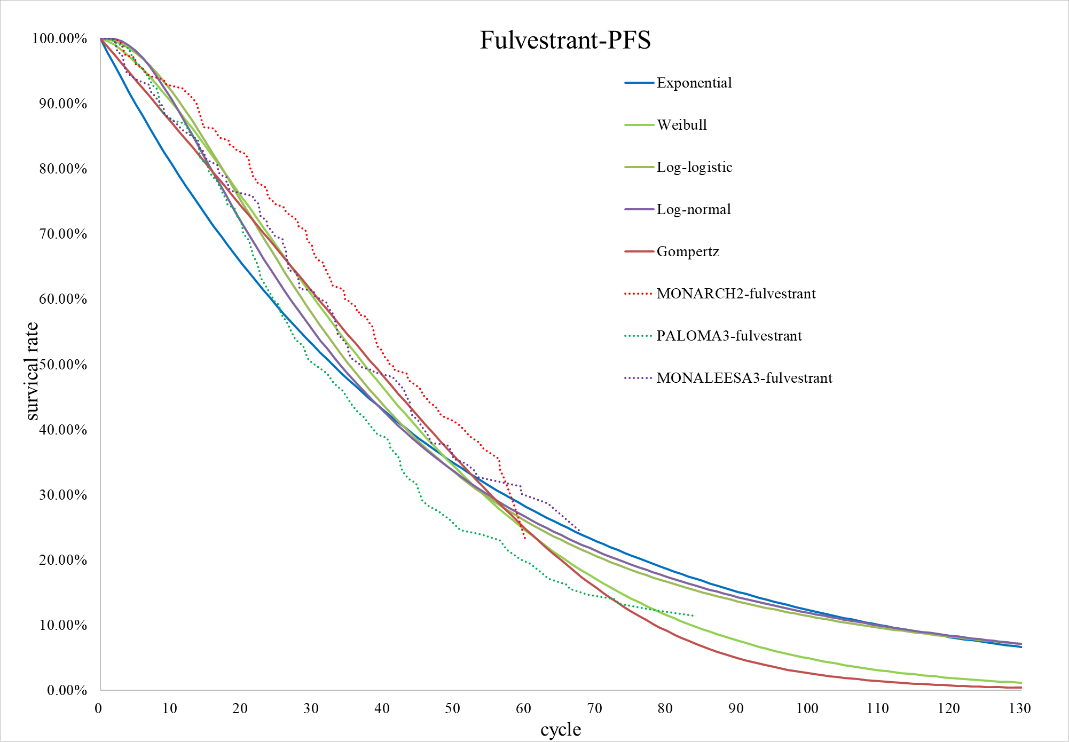


**Supplementary Figure 8**. The reconstructed Kaplan-Meier OS curves of fulvestrant monotherapy


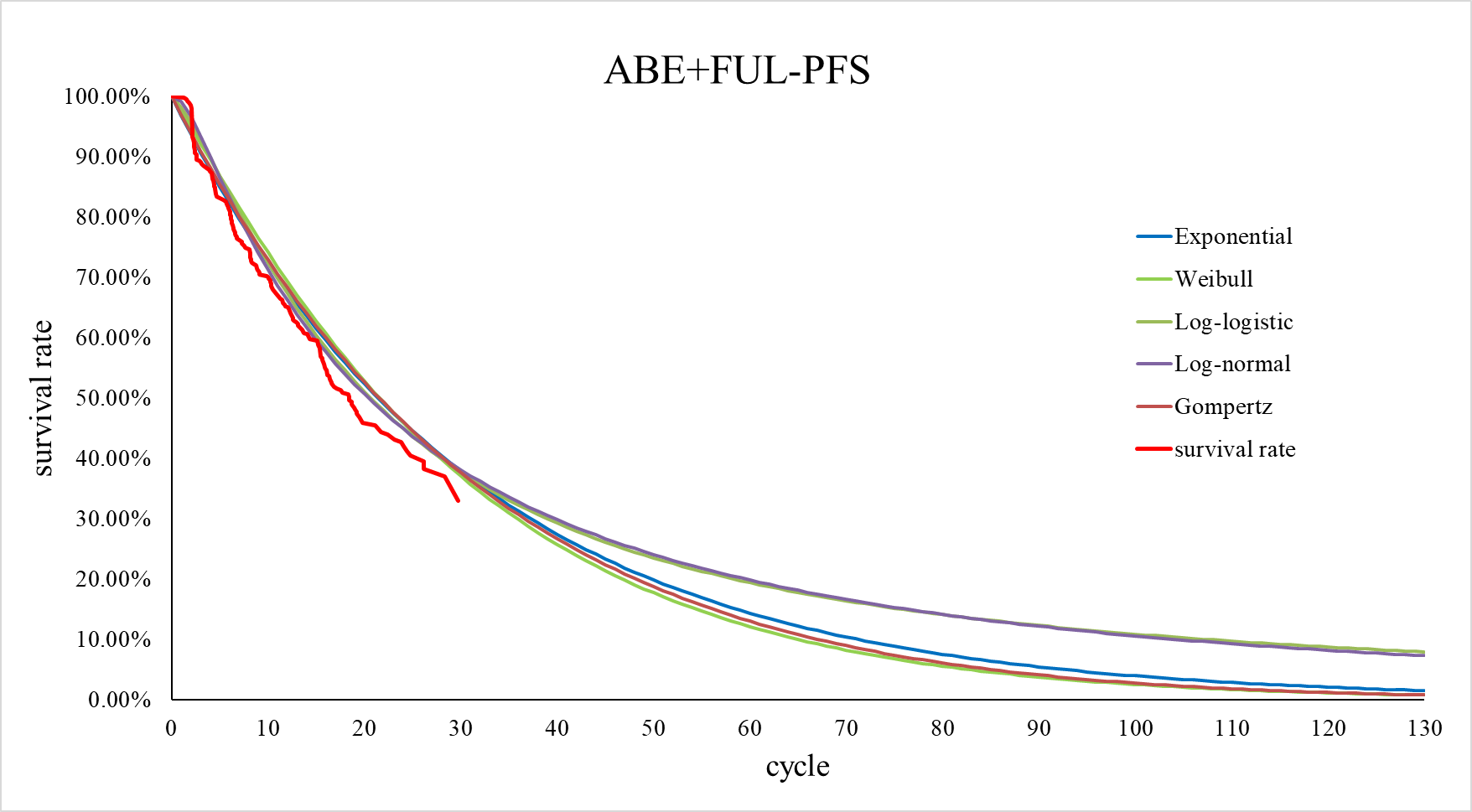

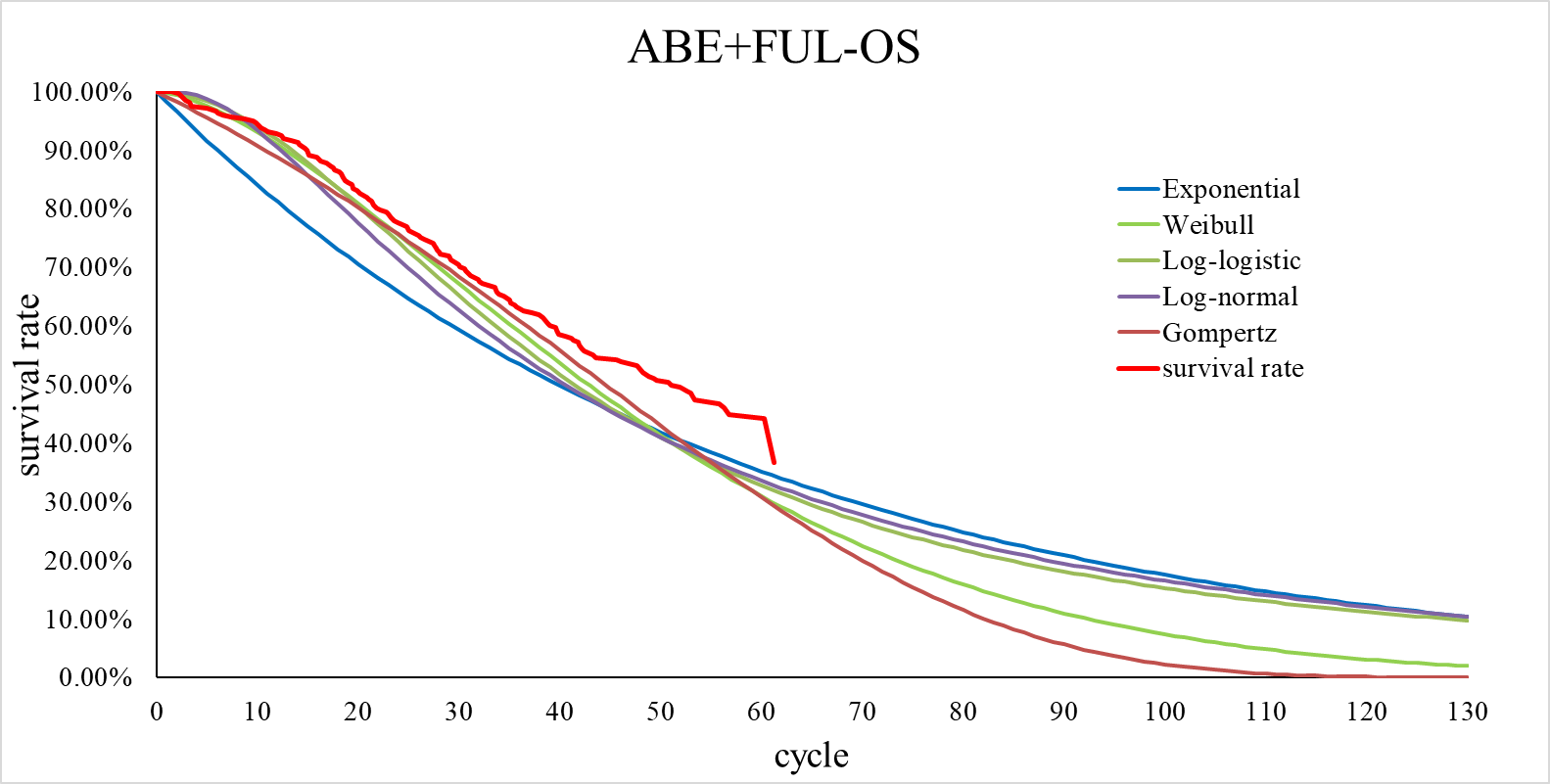
**Supplementary Figure 9**. The reconstructed Kaplan-Meier PFS curves of abemaciclib plus fulvestrant

**Supplementary Figure 10**. The reconstructed Kaplan-Meier OS curves of abemaciclib plus fulvestrant


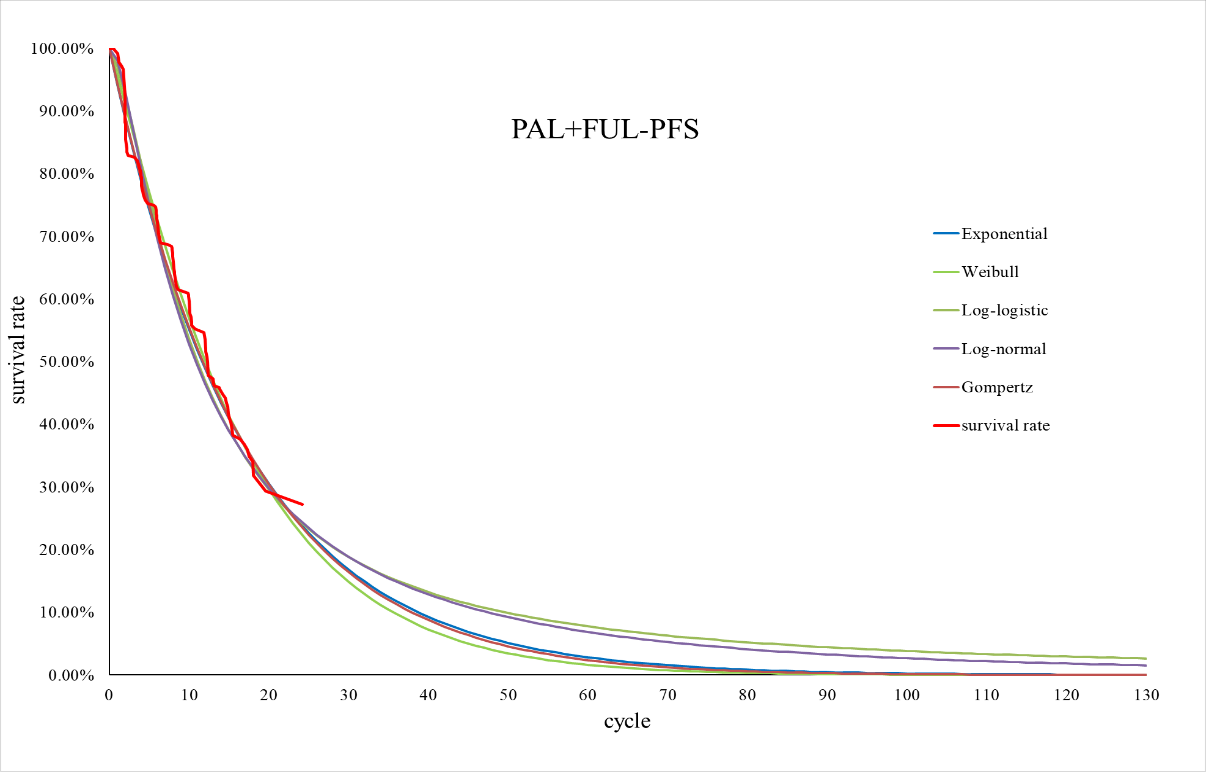

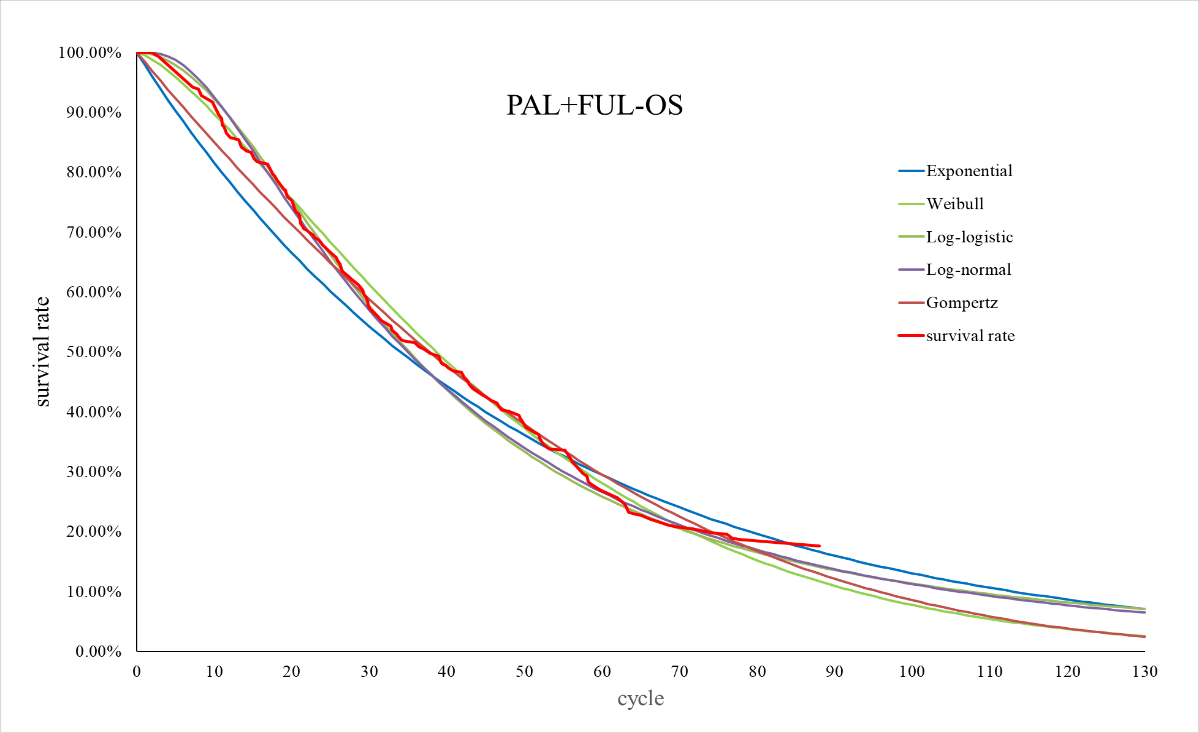
**Supplementary Figure 11**. The reconstructed Kaplan-Meier PFS curves of palbociclib plus fulvestrant

**Supplementary Figure 12**. The reconstructed Kaplan-Meier OS curves of palbociclib plus fulvestrant


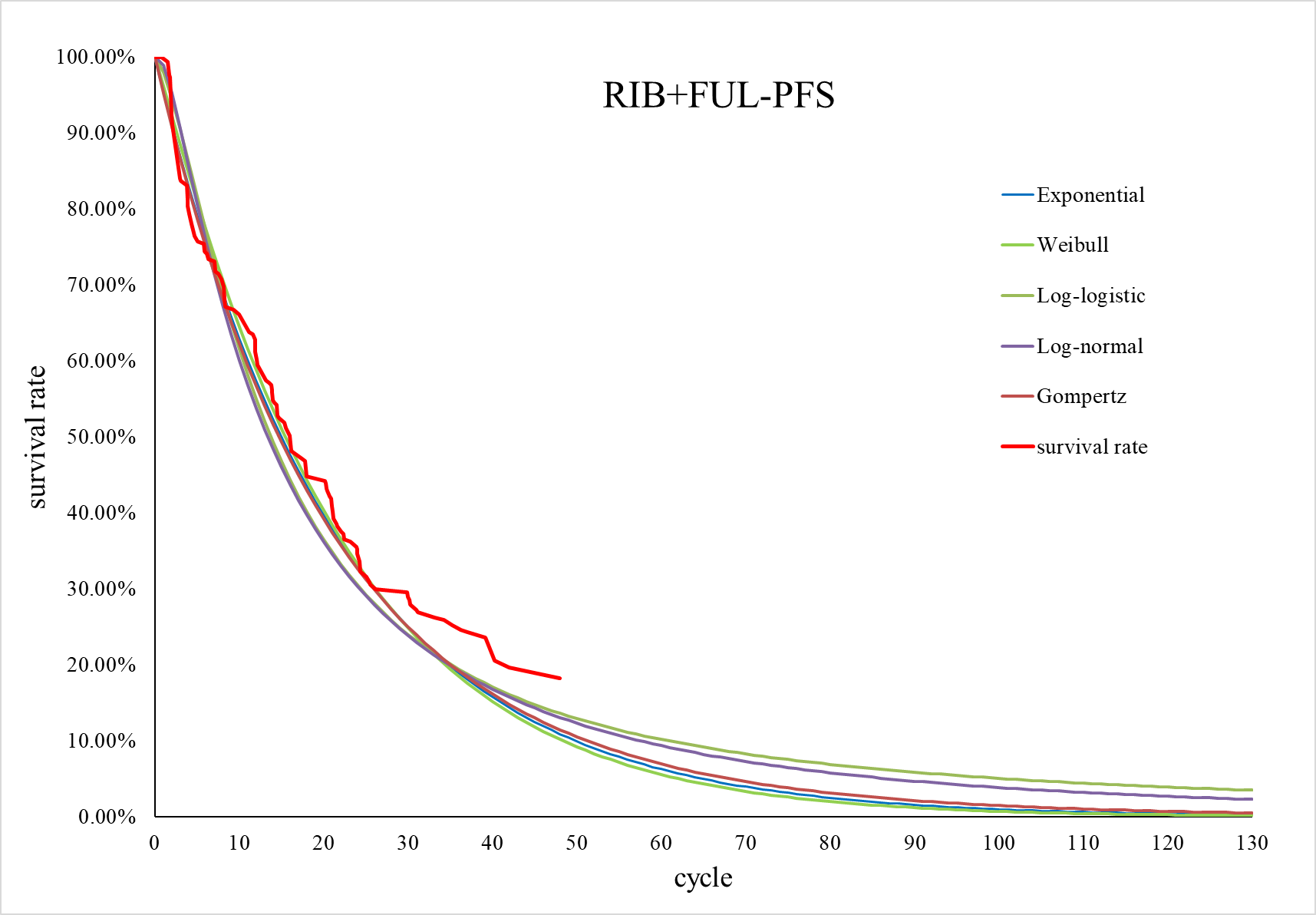

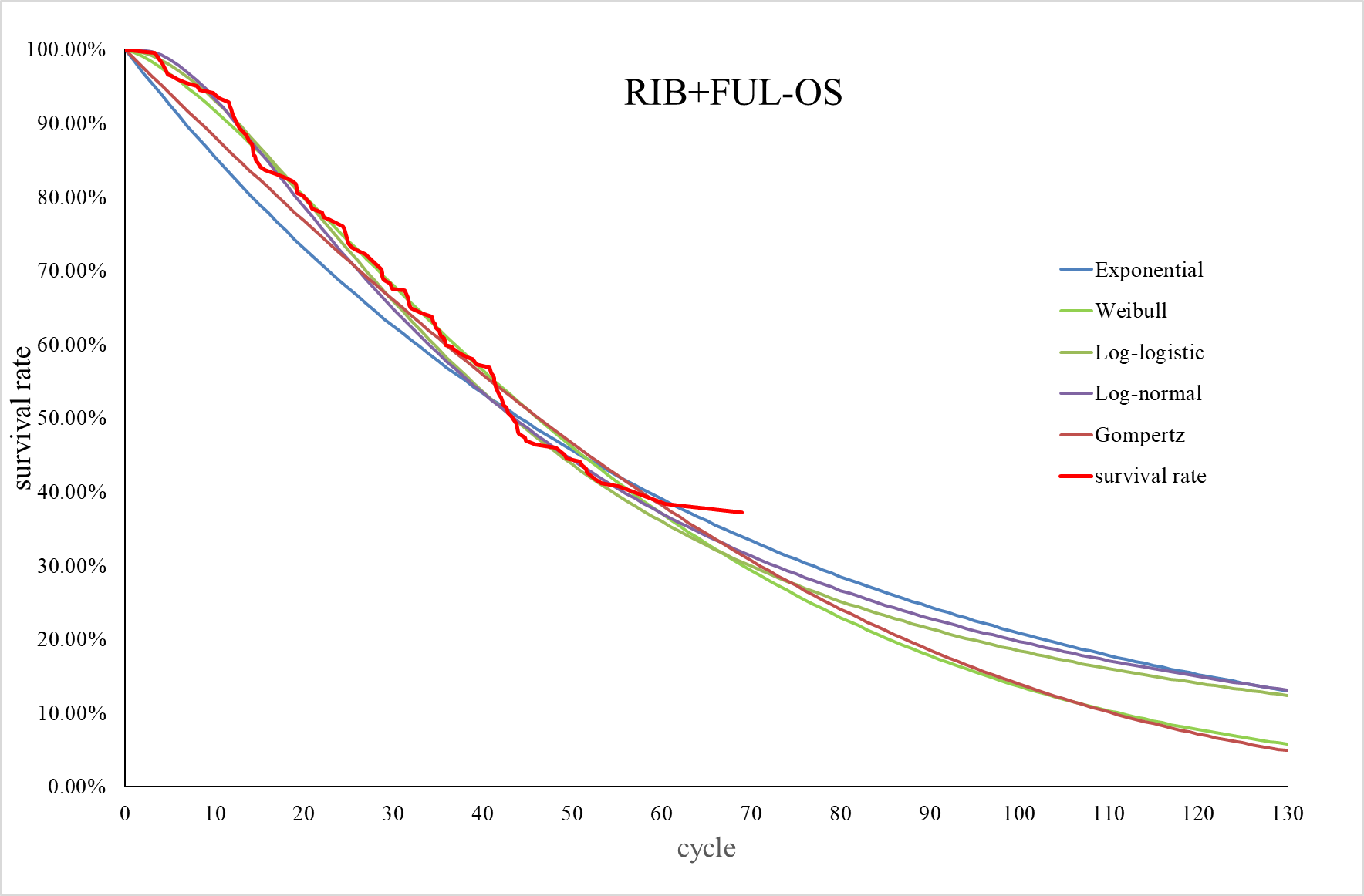
**Supplementary Figure 13**. The reconstructed Kaplan-Meier PFS curves of ribociclib plus fulvestrant

**Supplementary Figure 14**. The reconstructed Kaplan-Meier OS curves of ribociclib plus fulvestrant
